# Differential Distribution of the Ca (2+) Regulator Pcp4 in the Branchial Arches Is Regulated by Hoxa2

**DOI:** 10.1371/journal.pone.0063160

**Published:** 2013-05-09

**Authors:** Megan Anderson, Shilu Amin, Fabiana Luise, Leo Zeef, Nicoletta Bobola

**Affiliations:** 1 Faculty of Medical and Human Sciences, Manchester Academic Health Science Centre, University of Manchester, Manchester, United Kingdom; 2 School of Dentistry, Faculty of Medical & Human Sciences, Manchester Academic Health Science Centre, University of Manchester, Manchester, United Kingdom; 3 Faculty of Life Sciences, University of Manchester, Manchester, United Kingdom; 4 Centre for Endocrinology & Diabetes, Institute of Human Development, Faculty of Medical and Human Sciences, Manchester Academic Health Science Centre, University of Manchester, Manchester, United Kingdom; Instituto Gulbenkian de Ciência, Portugal

## Abstract

Branchial arches are externally visible tissue bands in the head region of all vertebrate embryos. Although initially formed from similar components, each arch will give rise to different head and neck structures. In a screen designed to characterize the molecular control of branchial arch identity in mouse, we identified Pcp4 as a second branchial arch-specific molecular signature. We further show that the transcription factor Hoxa2 binds to *Pcp4* chromatin and regulates *Pcp4* expression in the second arch. Hoxa2 is also sufficient to induce *Pcp4* expression in anterior first arch cells, which are Pcp4-negative.

## Introduction

Branchial arches are transient, repetitive structures of the vertebrate embryo, in which cells of the cranial neural crest and mesoderm are encapsulated by epithelia. There are five to six pairs of arches in amniotes, labeled according to their position along the embryo antero-posterior axis; the first corresponds to the most anterior arch (lying below the forebrain) and the sixth to the most posterior (close to the developing heart). Each of the arches shares a similar architecture and ability to form skeletal elements, their associated muscles, blood supply and nerves, but has its own molecular identity and contributes to diverse head and neck structures. Early development of the branchial arches is instructed by signaling molecules and transcription factors. Dlx transcription factors regulate proximo-distal patterning within each branchial arch [1]. Hox transcription factors control branchial arch identity [2]–[5]. A classic example of molecular control of inter-branchial arch identity concerns the first and the second branchial arch. The second arch is the most anterior of the branchial arches to express Hox genes, and mainly expresses *Hoxa2* and *Hoxb2*; the first arch does not express any Hox genes. Hoxa2 specifies second branchial arch (IIBA) identity: the second arch follows a first arch fate in the absence of Hoxa2, and IIBA-skeletal derivatives are replaced by typical first branchial arch (IBA) skeletal elements in a mirror image configuration [2]–[4].

Pcp4 is a small calmodulin (CaM)-binding protein. It affects the rates of association and dissociation of Ca (2+) from CaM and can attenuate the activity of a number of CaM-dependent enzymes, including CaM kinase II [6]–[8]. Initially identified as highly abundant in Purkinje cells, it is highly expressed in the developing neural system [9], [10] and has the ability to inhibit cell death [10] and to promote neurite outgrowth *in vitro* [11]. Changes in the synaptic plasticity of cerebellar Purkinje cells are observed in *Pcp4*-null mice [12]. Non-neural effects of Pcp4 have also been described, including a positive role in the osteoblastic differentiation of bone marrow stem cells [13] and in the differentiation of B cells [14].

To understand the molecular control of inter-branchial arch identity, we forced expression of *Hoxa2*, the main determinant of second arch fate, in first arch cells and analyzed changes in global expression. By intersecting the data obtained with related data sets, we uncover novel genes regulated by Hoxa2. In addition, we identify the gene encoding for Pcp4 as highly differentially expressed in first and second arch cells. We show that, while anterior first arch cells are Pcp4-negative, Pcp4 is enriched in second arch cells, and that *Pcp4* expression is directly controlled by the transcription factor Hoxa2 *in vivo*.

## Results and Discussion

**Figure 1:**
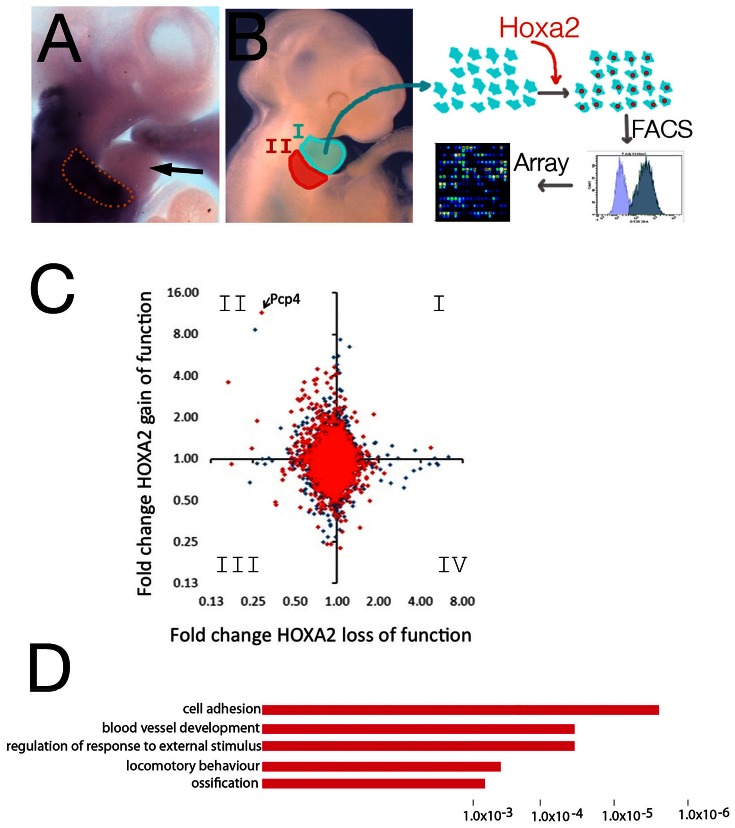
*Hoxa2* gain of function in IBA cells. A, In situ hybridization using *Hoxa2* probe shows *Hoxa2* is mainly expressed in the IIBA (enclosed in the dotted line) and expression is excluded from the IBA (arrow). B, Schematic representation of the experiment: cells isolated from IBA are grown *in vitro* and transfected with *Hoxa2-IRES-GFP* (or *GFP* alone, control). RNA is extracted from GFP-positive cells and analyzed by microarray. C, Pairwise comparison of microarray experiments for *Hoxa2* loss of function (x-axis) versus *Hoxa2* gain of function (y-axis). Data are plotted as fold change against control in each case (axes in logarithmic scale base 2). Genes in red are nearby a Hoxa2-bound region in ChIP-seq (closest two genes to Hoxa2-bound region were included). D, Functional annotation of genes responsive to *Hoxa2* gain of function only. The top over-represented categories are shown; the length of the bars corresponds to the P-values on the x-axis.

The transcription factor Hoxa2 specifies second arch identity: the presence of Hoxa2 in IIBA cells modifies the similar ground state shared by first branchial arch (IBA) and IIBA cells and instructs a second arch developmental fate (Fig. 1A) [2], [3], [15]. To understand Hoxa2 molecular control of branchial arch development, we forced the expression of *Hoxa2* in Hox-negative IBA cells (Fig. 1A). Cells isolated from the branchial arches maintain their molecular identity when grown *in vitro* [16]. First arch cells were transfected with *Hoxa2-IRES-GFP* or *GFP* alone and sorted using FACS. RNA was extracted from GFP-positive cells and the expression profile of *Hoxa2*-positive IBA cells was compared to control, Hox-negative cells, to identify differentially expressed genes (Fig. 1B). We identified 59 genes highly differentially expressed upon *Hoxa2* gain of function in IBA cells (fold change ≥2.5; pvalue <0.05), distributed in 36 upregulated and 23 downregulated genes (Table S1). We intersected the genes differentially regulated in *Hoxa2* gain of function in IBA cells with two complementary sets of data, the genes bound by Hoxa2 in the IIBA (Hoxa2 ChIP-seq) [17] and the genes differently regulated in wild-type versus *Hoxa2* loss of function mutant IIBA [17]; results are summarized in Table 1. We observed the largest overlap between genes upregulated in *Hoxa2* gain of function and downregulated in *Hoxa2* loss of function. This group, which contains genes activated by Hoxa2, is frequently associated with Hoxa2 bound regions (Fig. 1C, II quadrant; Table S2, segment S1). Conversely, most of the genes upregulated in the *Hoxa2* loss of function mutant (repressed by Hoxa2) are not affected by *Hoxa2* overexpression and are seldom associated to Hoxa2 binding events, suggesting these changes may be indirect effects of Hoxa2 absence (Fig. 1C, I and IV quadrant; Table S2, segment S5). Interestingly a total of 328 Affymetrix probes (corresponding to 255 unique genes) measured expression changes exclusively in *Hoxa2* gain of function, but not in the *Hoxa2* mutant IIBA (139 upregulated; 188 probes downregulated; Table S2, segment S2 and S7). They included genes displaying high expression changes upon *Hoxa2* gain of function. DAVID analysis [18] clustered the corresponding genes into functional categories closely related to the biological processes regulated by Hox proteins in embryonic development [19]–[21] (Fig. 1D). These changes may result from Hoxa2 functioning in a different biological context, despite the common developmental ground state of IBA and IIBA, and correspond therefore to non-physiological Hoxa2 targets. The IIBA expresses *Hoxb2* (and to a lesser extent *Hoxa1*), which may potentially compensate *Hoxa2* loss of function and mask changes in the expression of Hoxa2 targets. Gain of function of *Hoxa2* in IBA cells provides therefore a complementary system to identify Hoxa2 direct targets in branchial arches mesenchymal cells. Finally, few genes displayed changes in expression with the same sign in *Hoxa2* gain and loss of function (Table S2, segments S3 and S6).

**Table 1:** Expression changes in *Hoxa2* loss of function (LOF) and *Hoxa2* gain of function (GOF).

		LOF Down	LOF Up
	**Total**	77 (59)	30 (19)
**GOF Up**	151 (118)	10 (9)	1
**GOF Down**	191 (150)	3 (3)	0

The numbers of Affymetrix probesets with fold change > = 1.75 in either of these experiments are shown; the corresponding unique genes numbers are shown in brackets.

**Figure 2:**
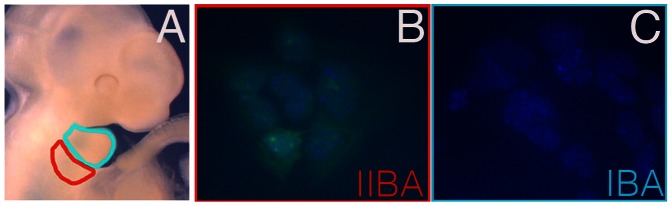
Pcp4 is specifically expressed in IIBA cells. A, Head region of a midgestation mouse embryo, with first and second arch highlighted in turquoise and red, respectively. B,C. Immunofluorescence using Pcp4 antibody (green) stains cells isolated from IIBA (B), but not IBA (C). Nuclear staining is blue (DAPI).

The gene encoding for Pcp4 displayed the highest fold difference in expression in first arch cells transfected with *Hoxa2*, with low to undetectable level of expression in GFP-positive first arch cells (fold change = 11.5; pvalue = 0.0005). Interestingly, expression of *Pcp4* was also highly different when comparing expression profiles of cells isolated from the first and the second arch (NB, data not shown). Since *Hoxa2* expression is sufficient to induce *Pcp4* expression in IBA cells, we asked whether IIBA cells, which are *Hoxa2*-positive, endogenously express *Pcp4*. We analyzed the expression of Pcp4 in branchial arch cells using immunofluorescence: Pcp4 was visualized only in IIBA cells, and was not present in cells derived from the IBA (Fig. 2A–C). We observed a similar distribution in *Pcp4* transcripts (Fig. S1).

**Figure 3:**
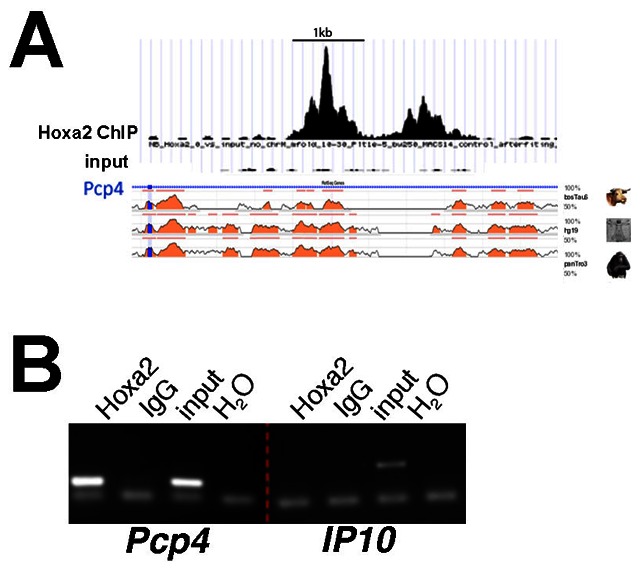
Hoxa2 binds *Pcp4 in vivo*. A, High enrichment of the first intron of the *Pcp4* gene compared to input (mm9, chr16∶96,717,332-96,718,404) in Hoxa2 ChIP-seq. Evolutionary Conserved Regions (ECRs) plot generated by the ECR Browser, comparing the genomic region bound by Hoxa2 between human, chimpanzee, and cow [29]. B, Conventional ChIP on IIBA chromatin confirms enrichment of Hoxa2 to *Pcp4*. *IP10* is a negative control gene. IgG is a non-specific negative control antibody.

A survey of Hoxa2 binding events in the IIBA [17] identified binding of Hoxa2 in the first intron of *Pcp4* (Fig. 3A). The corresponding peak is included in the top 1% most highly enriched regions after immunoprecipitation with Hoxa2 antibody, which is indicative of strong Hoxa2 binding. The summit region (200-bp window centered on ChIP-seq peak) contains Hox-Pbx and Meis motifs and six TAAT motifs, which correspond to Hoxa2 recognition site [17] (data not shown).

**Figure 4:**
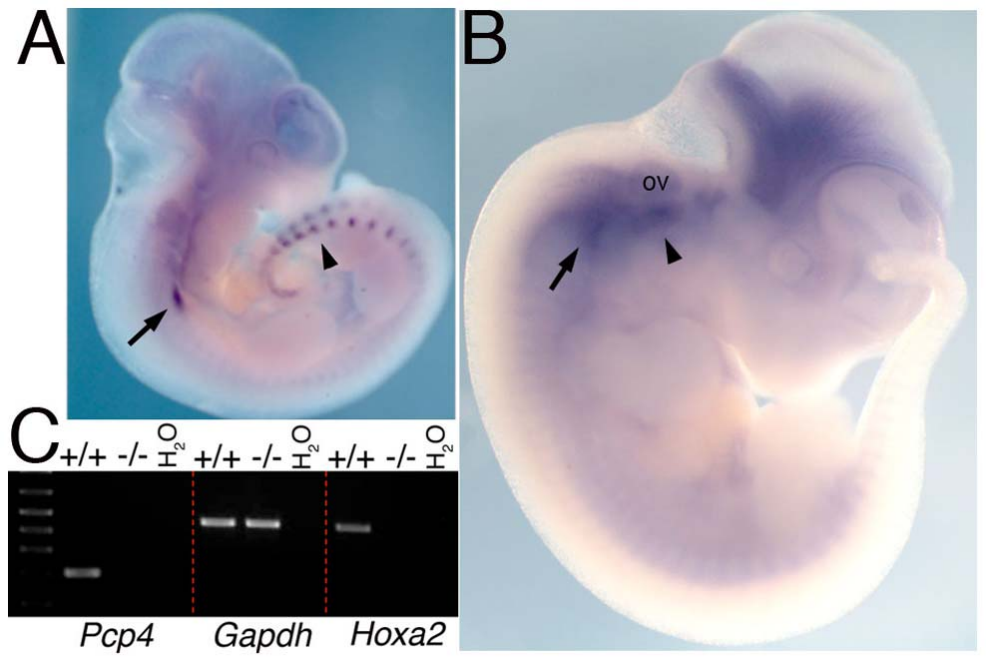
Hoxa2 regulates *Pcp4* in the branchial arches. A,B. Whole mount ISH on E10.5 (A) and E11.5 (B) wild-type embryos, using *Pcp4* probe. *Pcp4* is expressed in the cranial ganglia (arrow, A-B) and dorsal root ganglia (arrowhead in A). One day later *Pcp4* expression intensifies in the proximal area of the IIBA (arrowhead in B) in close proximity to the otic vesicle, and in the brain, and caudal expression disappears. C, Semiquantitative RT-PCR in wild type (+/+) and *Hoxa2* mutant (−/−) E11.5 IIBA cDNA. *Pcp4* is expressed in wild type and not *Hoxa2* mutant IIBA cells. *Gapdh* is a positive control gene and *Hoxa2* expression is confirmed in wild type and not *Hoxa2* mutant IIBA cells. Ov, otic vesicle.

We immunoprecipitated IIBA chromatin and found that the region bound by Hoxa2 on *Pcp4* intron was highly enriched after immunoprecipitation with a Hoxa2-specific antibody, but not with an unrelated antibody (Fig. 3B). These findings indicate that Hoxa2 is bound to *Pcp4 in vivo*. Next, we analyzed the expression of *Pcp4* in the developing embryo. At E10.5 *Pcp4* is expressed in the branchial arch region; this expression corresponds to the developing cranial ganglia [9] and does not appear to be specific for any of the BAs (Fig. 4A, arrow). In addition, *Pcp4* is strongly expressed in the dorsal root ganglia [9] (Fig. 4A, arrowhead), and in the developing eye. One day later (E11.5), the expression of *Pcp4* in the branchial area extends into the IIBA (Fig. 4B, arrowhead), and is contained within *Hoxa2* domain of expression in E11.5 embryos [17]. Strong expression is still observed in the cranial ganglia (Fig. 4B, arrow) and expression intensifies in the brain. We analyzed *Pcp4* expression in E11.5 IIBA isolated from wild type (+/+) and *Hoxa2* mutant (−/−). We found strong *Pcp4* expression in wild type IIBA, but no expression in *Hoxa2* mutant IIBA (Fig. 4C). These results indicate that the expression of *Pcp4*, observed in the IIBA, is dependent on Hoxa2.

Taken together, the observations that a) Hoxa2 is strongly bound to *Pcp4* intron in developing (E11.5) IIBA; b) Hoxa2 is sufficient to induce *Pcp4* expression in IBA mesenchymal cells; and c) Hoxa2 is required for expression of *Pcp4* in the IIBA, indicate that Hoxa2 is a main regulator of *Pcp4* in embryonic development. Additional experiments will be required to understand the function of Pcp4 in the development of the second branchial arch. Local modulation of calcium signal transduction is a key determinant of beak shape [22], linking control of Ca (2+) signaling to variations in craniofacial morphology. The control of *Pcp4* by Hoxa2, leading to its expression in the second arch, and the ability of Pcp4 to modulate Ca (2+) signaling suggest that acquiring IIBA identity may partly rely on a differential ability of IIBA cells to control Ca (2+) levels.

## Materials and Methods

### Mouse Lines, Cell Culture and Infection

*Hoxa2* mutant mice were described previously [2]. Branchial arches were isolated from E11.5 mouse embryos, obtained by CD1 mice time-matings. Branchial arches were dissociated into single cells using trypsin and a cell strainer (BD Falcon). A total of 200,000 cells were plated on a 24-well plate and grown in DMEM 10% FCS, at 37°C, 5% CO_2_. Ecotropic-Phoenix cells were transfected with Fugene and pMYs-IRES-GFP (Cell Biolabs, San Diego, CA) or pMYs-Hoxa2-IRES-GFP (containing Hoxa2 in front of the IRES), and branchial arch cells were infected using supernatants from Ecotropic-Phoenix packaging cells, supplemented with polybrene at 2 µg ml/1 final concentration. After 72 hours cells were sorted by retroviral gene expression of fluorescent proteins using flow cytometry. Animal experiments were carried out under ASPA 1986.

### Microarrays

RNA was extracted from sorted cells using Trizol (Life Technologies). Labeled targets were generated from total RNA obtained in two independent experiments, using the 3′ IVT Express Kit and hybridized to GeneChip Mouse Genome 430 2.0 arrays [17]. Background correction, quantile normalization, and gene expression analysis were performed using RMA in Bioconductor [23]. Principal component analysis (PCA) was performed with Partek Genomics Solution (version 6.5, Copyright 2010, Partek Inc., St. Charles, MO, USA). Differential expression analysis was performed using Limma using the functions lmFit and eBayes [24]. The microarray expression data has been submitted to ArrayExpress (ArrayExpress accession: E-MEXP-3837).

### Immunofluorescence

Branchial arch cells were isolated as described above and grown on 8-well culture slides (BD Falcon) for 24 hours, fixed and incubated with Pcp4 antibody (Sigma) diluted 1∶200 and visualized with AlexaFluor 488 goat anti-rabbit (Invitrogen).

### Chromatin Immunoprecipitation (ChIP)

ChIP was performed as described [25] using IIBAs isolated from E11.5 CD1 mouse embryos (24 pairs). The summit of Hoxa2-bound region on *Pcp4* gene [17] was amplified using forward 5′-ACTCTGTCAGAGCAATTACCA-3 and reverse 5′-CTTGTAGGCACTAACTGGGA-3 primers.

### Expression Analysis

Second branchial arches of E11.5 embryos from *Hoxa2^+/−^* intercrosses were dissected out and snap-frozen in dry ice. After genotyping the embryos, pools were made with the wild type and *Hoxa2^−/−^* branchial arches and total RNA was extracted using Trizol. RT-PCR was performed as described [26], and cDNA was subjected to 24 and 28 cycles of amplification using Hoxa2 primers [25] and *Pcp4* F 5′-ATGAGTGAGAGACAAGTGCC’-3; *Pcp4*R 5′-CTAGGACTGTGATCCTGCCT-3′, respectively. Whole mount *in situ* hybridization was performed as described [27], using *Hoxa2* [28], and *Pcp4* probe, amplified from IIBA cDNA using the primers above.

## Supporting Information

Figure S1 **Differential** ***Pcp4*** **expression in the first and second branchial arch.** Semiquantitative RT-PCR on cDNA isolated from first (I) and second (II) arch. *Pcp4* is detected in IIBA and not IBA. *Gapdh* is a positive control gene; H_2_O is the negative control, using both *Pcp4* and *Gapdh* primers.
(JPG)

Table S1 **Top regulated genes in** ***Hoxa2*** **gain of function in IBA cells.** Genes are ranked using fold changes. The following cut-offs were applied to the probe sets: average fold difference ≥2.5; P value ≤0.05.
(XLS)

Table S2 **Microarray data expressed as fold change and p values for the** ***Hoxa2*** **loss of function (LOF) and** ***Hoxa2*** **gain of function (GOF) microarray experiments.** Affymetrix probesets are only included if they have a fold change > = 1.75 in either of these experiments. Additional information shows if a gene passes combined filter thresholds of + or − 1.75; 1 indicates yes and 0 indicates no. Also indicated is whether the gene is located close to a Hoxa2-bound region in ChIP-seq binding site (where “close” is defined as being one of the two closest genes to a ChIP-seq peak).
(XLS)
