# Supplementary figures and images for: Differential Distribution of the Ca (2+) Regulator Pcp4 in the Branchial Arches Is Regulated by Hoxa2
Source: PLoS One. 2013 May 9;8(5):e63160. doi: 10.1371/journal.pone.0063160 (PMC3650044; doi:10.1371/journal.pone.0063160)

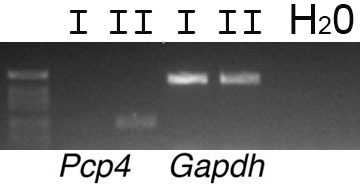

Supplement: Figure S1 — Differential Pcp4 expression in the first and second branchial arch. Semiquantitative RT-PCR on cDNA isolated from first (I) and second (II) arch. Pcp4 is detected in IIBA and not IBA. Gapdh is a positive control gene; H2O is the negative control, using both Pcp4 and Gapdh primers. (JPG) [file pone.0063160.s001.jpg]
